# Phenotypic and Functional Heterogeneity of Monocyte Subsets in Chronic Heart Failure Patients

**DOI:** 10.3390/biology11020195

**Published:** 2022-01-26

**Authors:** Aušra Mongirdienė, Julius Liobikas

**Affiliations:** 1 Department of Biochemistry, Medical Academy, Lithuanian University of Health Sciences, LT50161 Kaunas, Lithuania; 2 Laboratory of Biochemistry, Neuroscience Institute, Lithuanian University of Health Sciences, LT50162 Kaunas, Lithuania

**Keywords:** monocyte subset, heart failure, inflammation, cytokine, macrophage

## Abstract

**Simple Summary:**

A long-term condition known as chronic heart failure (CHF) is an ongoing difficulty of the heart in pumping blood enriched in oxygen and required nutrients around the body’s tissues. CHF pathogenesis is associated with various causes, and inflammation is one of the most important factors promoting the condition. In addition, monocytes, a group of cells present in the blood and infiltrating tissues, are known to participate in both pro- and anti-inflammatory processes and thus affect myocardial remodeling over time. The aim of this work was to review current studies on the function of monocyte subsets in different types of CHF with preserved and reduced left ventricle ejection fractions and to discuss the relationship of monocyte subsets to inflammatory markers. It is expected that a deeper view into CHF pathogenesis could stimulate the search for and development of individualized therapies.

**Abstract:**

Chronic heart failure (CHF) results when the heart cannot consistently supply the body’s tissues with oxygen and required nutrients. CHF can be categorized as heart failure (HF) with preserved ejection fraction (HFpEF) or HF with reduced ejection fraction (HFrEF). There are different causes and mechanisms underlying HF pathogenesis; however, inflammation can be regarded as one of the factors that promotes both HFrEF and HFpEF. Monocytes, a subgroup of leukocytes, are known to be cellular mediators in response to cardiovascular injury and are closely related to inflammatory reactions. These cells are a vital component of the immune system and are the source of macrophages, which participate in cardiac tissue repair after injury. However, these monocytes are not as homogenous as thought and can present different functions under different cardiovascular disease conditions. In addition, there is still an open question regarding whether the functions of monocytes and macrophages should be regarded as causes or consequences in CHF development. Therefore, the aim of this work was to summarize current studies on the functions of various monocyte subsets in CHF with a focus on the role of a certain monocyte subset in HFpEF and HFrEF patients, as well as the subsets’ relationship to inflammatory markers.

## 1. Introduction

Cardiovascular diseases (CVDs) are a major cause of mortality worldwide, and the prognosis of such diseases remains poor. The 5-year survival rate for CVDs is about 50% after the diagnosis of heart failure (HF) [1]. Moreover, the number of patients diagnosed with HF is increasing, and this number is predicted to rise to more than 8 million by 2030, according to the American Heart Association’s 2017 Heart Disease and Stroke Statistics Update [2].

Chronic heart failure (CHF) is a condition in which the heart cannot continually supply the body’s tissues with the proper amount of oxygen and nutrients. There are different causes and mechanisms of CHF pathogenesis. Thus, depending on the pathogenesis, heart failure is categorized as either HF with preserved ejection fraction (HFpEF) or HF with reduced ejection fraction (HFrEF). HFpEF means that the patient’s heart extrudes close to the normal amount of blood (≥50% of the left ventricle ejection fraction (LVEF)) to the tissues, whereas HFrEF means that the amount of blood is lower than normal, i.e., <50% of LVEF [1]. HFpEF contributes to about 50% of the total HF population [1]. Notably, the one-year mortality of HFrEF was shown to be higher than that of HFpEF, but this varies between studies (8.2–21% vs. 2.7–13%) [3]. Although the exact sequence of events contributing to the development and progress of HF remains to be elucidated, HFrEF is known to be the outcome of myocardial ischemia and infarction. Notably, HFpEF is associated with older age, dysregulated metabolism and chronic hypertension, contributing to oxidative stress and myocardial dysfunction [4]. It was also suggested that during early HF stages, systemic inflammation can induce endothelial dysfunction, consequently promoting the invasion of pro-inflammatory cells such as monocytes into the heart tissue and contributing to the increased stiffness of the myocardium [5]. Altered levels of matrix metalloproteinases and a modified composition of the myocardial extracellular matrix can further contribute to the progression of myocardial dysfunction [6,7]. Thus, inflammation is regarded as one of the factors promoting HF in both HFrEF and HFpEF [8].

Consequently, efforts to reduce inflammation in blood vessel walls and modulate the anti-inflammatory processes in the monocyte/macrophage system could represent a promising therapeutic approach [9]. Monocytes, a subgroup of leukocytes (white blood cells), are known to be cellular mediators in response to cardiovascular injury and are closely related to inflammatory reactions [10,11]. These cells are a critical component of the innate immune system and are the source of many vital elements in the immune system, such as macrophages, which sense and respond to pathogens and other environmental challenges, as well as participate in tissue repair after injury. Monocytes play a role in both the pro- and anti-inflammatory processes that take place during an immune response in tissue repair and continue even after the acute phase [7,10]. Recently, it was demonstrated that monocytes are not homogenous as previously thought and thus can present different functions under different CVD conditions [11]. Additionally, HFpEF can change into HFrEF. Hence, it is important to explore the pathological mechanisms of early diastolic dysfunction, such as monocyte activity, phenotype, and function. Therefore, in this work, we aimed to summarize current studies on the functions of various monocyte subsets in CHF with a special focus on the importance of a monocyte subset in HFpEF and HFrEF patients, as well as the relationship of subsets to inflammatory markers. More accurate knowledge about the pathogenesis of CHF could aid in the search for individualized therapies targeted at stopping harmful myocardial remodeling, thereby improving the patient’s heart function.

## 2. Nomenclature of Monocyte Subsets and Their Formation

Monocytes are the largest agranular leukocytes and are called circulating mononuclear phagocytes. Upon entering tissues, monocytes undergo morphological and functional changes and are then identified as macrophages. Both monocytes and activated macrophages, together with their cytokines, are essential to inflammation and sustain tissue responses that lead to chronic inflammation [9]. Monocytes are grouped according to the relative expression levels of their CD14 and CD16 surface proteins and chemokine receptors, as well as their phagocytic activity [11]. No expression is defined as “−”, moderate expression is defined as “+”, and high expression is defined as “++” [12]. Monocyte subsets also differ in their combinations of adhesion molecules and chemokine receptors, upon which different monocyte functions depend. It is also known that combinations of adhesion molecules and chemokine receptors can vary during the inflammation process [13].

According to the allotment renewed in 2017 by the Nomenclature Committee of the International Union of Immunologic Societies (NCIUIS), there are three distinct subsets of monocytes, defined as classical (CD14^++^/CD16^−^, or Mon1), intermediate (CD14^++^/CD16^+^, or Mon2), and non-classical (CD14^+^/CD16^++^, or Mon3), in the blood of healthy adults [11]. Notably, there are reports [13,14,15,16,17,18] suggesting that an additional CD14^+^/CD16^+^ monocyte subset could be distinguished. There are also suggestions that this subset could be related to the intermediate subset [19]. However, this proposition needs further clarification. Thus, despite discrepancies in the literature on the nomenclature of human monocytes, in this article, the classification system approved by the NCIUIS is followed. Moreover, the available literature data indicate variations in the levels of secreted cytokines and surface markers among and within individual monocyte subsets obtained from fresh and cryopreserved bone marrow and blood of human and mouse origin. Therefore, the most strongly overlapping results obtained after the investigation of the fresh monocytes from human blood are summarized. The summarized results on monocyte subsets, certain surface markers, produced specific cytokines, physiological functions, distribution in the blood, and implicit locations of subset formation are presented in Table 1.

**Figure 1:**
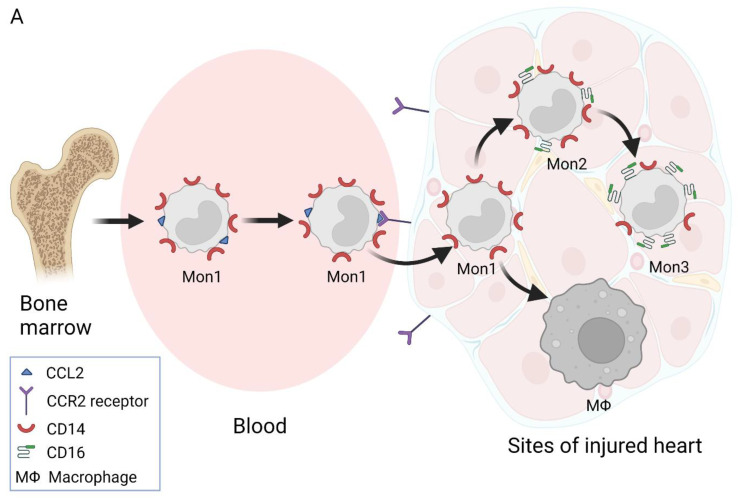

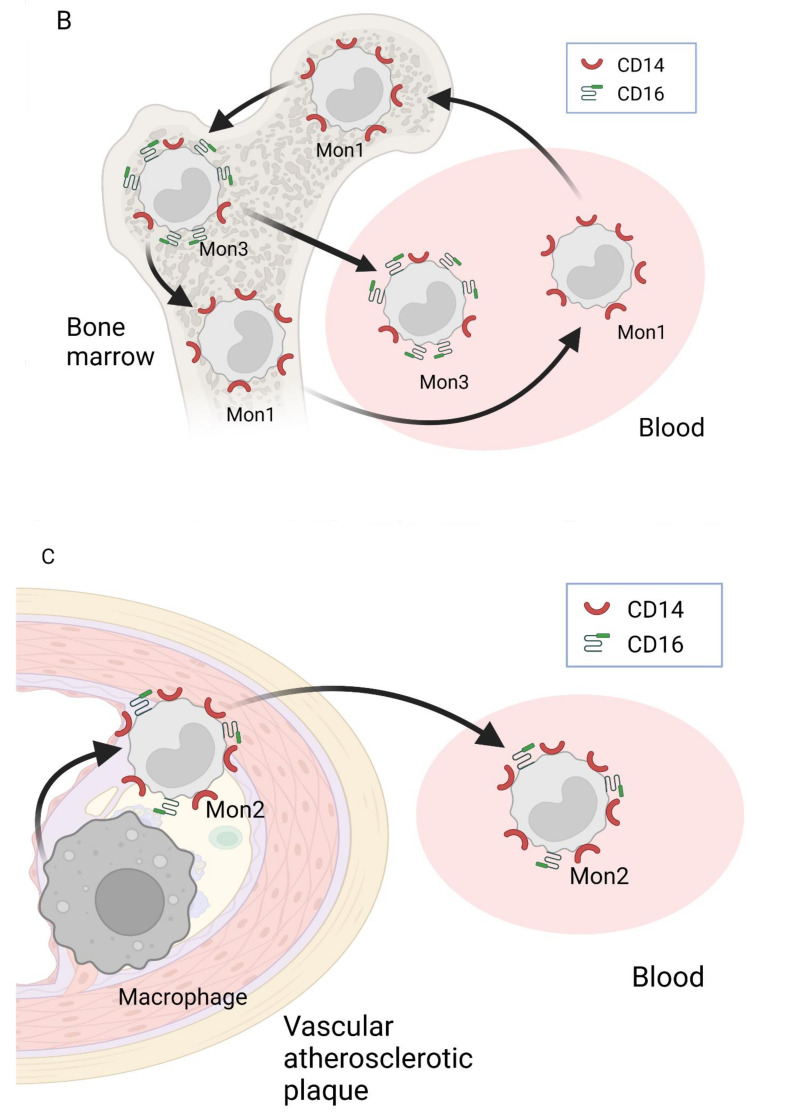
Scheme of putative monocyte subset formation [20,42,45,49,50] (created with BioRender.com on 18 January 2022). (**A**) One theory states that human monocytes mature in the bone marrow and are subsequently released into circulation as Mon1 monocytes. These monocytes migrate to sites of injury in a CCR2 (chemokine (C-C motif) receptor)-dependent manner and differentiate into macrophages. Progressively, the Mon1 monocytes give rise to the Mon3 subset through the Mon2 subtype of monocytes. (**B**) Mon1 monocytes can become both Mon2 and Mon3 subsets or give rise to Mon3 monocytes through the Mon2 subset. It is thought that a portion of Mon1 monocytes from the circulatory system or tissues that return to the bone marrow can also be converted into the Mon3 subset. (**C**) The third possibility suggests that Mon2 monocytes can be formed from macrophages in vascular atherosclerotic plaques and released into the circulatory system. Notably, there is scientific evidence for the co-existence of all three monocyte subsets in bone marrow. Mon1: classical monocytes; Mon2: intermediate monocytes; Mon3: non-classical monocytes.

Different monocyte subsets also differ in their origin. Mon1 monocytes are synthesized in the bone marrow and, from there, enter the bloodstream (Figure 1A). In the literature, the average lifespan of Mon1 is reported to be 1.0 (SD = 0.26) day [42]. Most Mon1 monocytes leave the circulation or die, whereas the remaining cells transform into the Mon 2 subset [42]. The average lifespan of Mon2 monocytes was found to be 4.3 (0.36) days, and all of them transform into Mon 3 monocytes. Notably, the average lifespan of the Mon3 subset was reported to be the longest, i.e., 7.4 (0.53) days [42]. It is also thought that Mon2 monocytes may convert into Mon1 monocytes after entering the blood [20]; alternatively, the Mon1 monocytes released from bone marrow may differentiate into both Mon2 and Mon3 monocytes [19]. Thus, there are several theories for the origin of Mon3 [20,42,43,44,45,46,47,48,49,50] (see Figure 1). The possibility that different monocytes are formed through all of the aforementioned ways cannot be ruled out.

However, it is not completely clear what determines the development of different monocyte subsets. Infections and human chronic or metabolic disorders could stimulate the production or prevalence of certain subsets. For instance, obesity has been found to cause the domination of Mon2 and Mon3 subsets over Mon1 [42,46]. Accordingly, obesity was previously characterized by a higher number of monocyte-derived adipose tissue macrophages in both mouse models and humans [51,52]. It is also worth noting that caloric restriction demonstrated favorable effects in several chronic metabolic disorders and CVD [48,53]. In addition, short-term fasting reduced the counts of all monocytes in healthy human subjects [54]. Moreover, it was found that certain growth factors can determine the formation of a particular subset of monocytes [54].

Thus, although it is agreed that Mon1 monocytes originate from precursors in bone marrow, the origins of other monocyte subsets and regulatory mechanisms leading to the formation of a particular monocyte subset depend on the cellular environment and still need further experimental analysis.

## 3. Involvement of Different Monocyte Subsets in the Inflammatory Processes

Monocytes are a part of the mononuclear phagocytosis system and are essential for the immune system [40]. Monocytes protect tissues from harmful pathogens via the direct removal of pathogens by phagocytosis and are also considered the major source of various cytokines and precursors of macrophages and dendritic cells. Mon1 monocytes take part in the immune response through releasing cytokines and via differentiation into macrophages and dendritic cells [40]. Notably, under pro-inflammatory conditions, stimulated macrophages can be not only beneficial but also harmful, as such macrophages can contribute to the inflammation associated with chronic diseases [55].

The main function of Mon1 is the initiation of an inflammatory response and phagocytosis [39,40]. Mon1 monocytes can enter non-inflamed tissues, where they express major histocompatibility complex MHC II [40]. During bacterial infection, Mon1 monocytes are deployed at sites of inflammation, where they recognize and phagocytize pathogens and secrete high levels of pro-inflammatory cytokines (IL-1β, IL-6) and low levels of anti-inflammatory cytokines (IL-10) by secreting monocyte chemotactic protein-1 (MCP1) and CCL2 [56]. Mon1 monocytes attract other immune cells to regulate the inflammatory response. It is known that Mon1 monocytes have higher peroxidase activity, higher ROS production, and are also linked to a more pronounced expression of macrophage antigen-1 (Mac-1) and scavenger receptors SR-A1 (CD204) and SR-A2 (macrophage receptor with collagenous structure, MARCO), as well as stronger binding to plasma low-density lipoprotein (LDL) compared to the Mon3 subtype [39,56,57,58]. Consequently, Mon1 monocytes are phagocytically more active than Mon3 cells and actively occur at the initiation, development, and resolution stages of inflammation in tissues [57,58,59,60,61].

Mon2 monocytes are involved in inflammatory processes through antigen presentation, cytokine secretion, the regulation of apoptosis, and angiogenic activity [39,40]. These monocytes express a stronger pro-inflammatory capacity than the Mon3 subset since the cells produce higher levels of ROS, TNF-α, IL-1β and IL-6, and CCL3 and express the highest levels of antigen-presentation-related molecules to restore the damaged tissue [36,62,63,64]. Thus, it seems that the prolonged activity of Mon2 becomes harmful. For instance, the intermediate subset was associated with chronic vascular and endothelial damage and atherosclerosis [28,36,39,57,58,59,60,61,62,63,64]. Therefore, Mon 2 may be involved in the maintenance of chronic inflammation, leading to harmful remodeling of cardiac tissue [21]. However, the reasons for the persistent activity of Mon2 and, consequently, for their involvement in chronic inflammation, remain unclear.

The third subset of monocytes (Mon3) produces significantly lower levels of pro-inflammatory cytokines compared to Mon1 [21]. Moreover, Mon3 occur in complement and Fc γ receptor-mediated phagocytosis and neutrophil adhesion at the endothelial interface [39,62]; thus, Mon3 monocytes can be regarded as endothelial patrols [65]. Both Mon1 and Mon3 monocytes are found in the coronary vasculature of healthy people. However, Mon1 monocytes are shown to circulate rapidly, whereas Mon3 monocytes circulate more slowly, crawling along the endothelium [66] (Table 1).

The debate about sex as an additional risk factor for HF development has also been reported [12]. The factors associated with HF in men were age, ischemic heart disease, and comorbidities, whereas in women, a combination of lifestyle factors, age, body mass index, hypertension, and atrial fibrillation was found to be of greatest importance [12]. It seems that HFrEF as a main result of ischemic heart disease would dominate in men, while HFpEF as a result of increased body mass index and hypertension would dominate in women. However, to the best of our knowledge, there is no scientific evidence to support this proposition.

Estrogens are reported to decrease monocyte levels and the monocyte secretion of IL-6 and IL-12 [67]. The onset of menopause alters the levels of monocytes to concentrations comparable to those observed in males [67]. However, publications that confirmed differences in monocyte subsets between men and women (both healthy and HF) were not found.

Notably, upon entry into tissues, monocytes can develop into two additional types of cells: dendritic cells (Dsc) [68,69] and macrophages [14]. However, there are indications that Mon 1 monocytes are strongly involved in the transformation into macrophages but not into Dsc [66]. It was acknowledged that monocytes, depending on the nature of their surrounding cytokines, differentiate into two main macrophage subsets that can be distinguished by their origin, localization, and pro- or anti-inflammatory functions [55]. Thus, M1 or classically activated macrophages, which are regarded as pro-inflammatory macrophages, arise in response to IFN-γ and TNF-α secreted by lymphocyte T helper-1 (Th1) [45,70], whereas the formation of M2 or anti-inflammatory macrophages is stimulated by IL-4 and IL-13 released from lymphocyte T helper-2 (Th2) [55,71,72]. DSc and macrophages are not the subject of this article; therefore, this topic was not covered in the present review.

In summary, different monocyte subsets differ in their surface markers, the production of various cytokines and, consequently, their functions. Mon1 monocytes are involved in phagocytosis, innate immune responses and migration within tissues. Furthermore, these monocytes can differentiate into DSc and, depending on the environment, into pro-inflammatory (M1) or reparative anti-inflammatory (M2) macrophages, thereby playing an integral part in shaping inflammation and its resolution in tissues. Mon2 monocytes are involved in the regulation of apoptosis and show angiogenic activity, whereas Mon3 monocytes can be regarded as endothelial patrols that also take part in immune maintenance. Notably, the monocyte subsets presented and discussed in this work use the most common classification system. However, further categorization into transcriptionally distinct subsets is currently under way.

## 4. Monocytes in CHF

### 4.1. The Distribution of Monocytes in CHF

The roles of monocytes under different CHF conditions are complex and, depending on the different monocyte subset number, may encompass inflammatory processes leading to tissue damage or repair [73,74,75,76,77]. Articles investigating monocyte subset (CD14^++^; CD16^−^, CD14^++^; CD16^+^, CD14^+^; CD16^++^) distribution in CHF patients have been summarized. However, works in which monocyte subsets were classified differently are not analyzed in this paper.

Several studies have demonstrated that Mon1 is the predominant subset in HF (87–48%), followed by the Mon2 (5–44%) and Mon3 subsets (7.1–8.4%) [27,73,77,78,79]. Thus, the data indicate a significant expansion of the Mon2 subset in HF patients when compared to healthy controls (see also Table 2). Another study examined CHF patients with idiopathic dilated cardiomyopathy (65% of the investigated population) and ischemic heart disease (the remainder of the population; in total, n = 20) and found a higher total leukocyte count and a higher absolute monocyte count in the CHF group compared to healthy people, with no differences in the monocyte subset ratio (but not the cell count) between healthy and diseased people [77]. However, under ambulatory-treated HF conditions, the Mon1 and Mon2 subsets (50.0 ± 17.2% and 42 ± 17.2%, respectively) outnumbered the Mon3 monocytes (8.1 ± 4.0%) [74]. In contrast, the Mon1 subset significantly prevailed over the other two subsets in healthy people (see Table 2). Moreover, the proportion of Mon2 monocytes in HF was clearly increased when compared with the proportion in healthy people. Notably, the ratio of different monocyte subsets in HF varies and may be dependent on different CHF conditions and hemodynamic changes [27,77]. For example, CHFrEF can be associated with an increase in the proportion of the Mon3 subset when compared to healthy people and after acute exercise [6,27,77,79], while the Mon2 subset was found to be the most abundant in stable CHF, where patients were not classified according to LVEF [27].

Moreover, several studies found that increased levels of the Mon2 subset and decreased levels of the Mon 1 subset in CHF patients were related to HF severity, which was defined as a New York Heart Association (NYHA) functional class advancement including a reduction of LVEF [78]. Notably, several studies did not find a correlation between the monocyte subset percentage and NYHA functional class or LVEF [73,79]. In addition, the Mon2 levels directly correlated with the C-reactive protein (CRP, which reflects increased systemic inflammation) level and neutrophil count [78]. Recently, it was also found that the Mon2 count was the highest in CHF patients who died [73]. Older deceased patients were characterized by a worse NYHA functional class and contained a higher amino-terminal fragment of pro-B-type natriuretic peptide (NT-proBNP) level [73]. Moreover, the NYHA functional class correlated with the total monocyte count and percentage, and the CRP concentration correlated with the NT-proBNP level in HFrEF [80,81]. Therefore, it can be presumed that the more affected the heart muscle is, the more strongly the pro-inflammatory environment is related to the predominance of the Mon2 subset in patients with CHF (see Table 2). Notably, elevated levels of Mon2 monocytes were also found among HF patients, in which men with chronic HF and ischemia as a cause of disease represented the majority of the cohort [73]. Again, these results are supported by the fact that Mon2 monocytes express a higher pro-inflammatory capacity than the Mon3 subset [62]. Thus, it can be assumed that an increasing amount of Mon2 monocytes within damaged cardiac tissue is required for the earliest steps in wound healing [73]. However, Mon2 persistence exceeding the initial repair process could lead to longer-term inflammation-related deleterious effects in healthy remote myocardial areas. This observation may explain the worse prognosis among HF patients with increased levels of Mon2 monocytes [73]. Another study [82] also reported a link between the amount of the Mon2 subset and poorer prognosis in acute HF patients.

For the sake of completeness, it is worth mentioning a study that did not find any correlation between the percentage of the Mon2 subset or the number of Mon2 cells/μL and the NYHA functional class, LVEF, or estimated glomerular filtration rate, except for a weak statistically significant inverse correlation between the percentage of Mon2 monocytes and LVEF (r = −0.14) in ST-Segment Elevation Myocardial Infarction (STEMI) patients [83].

It was demonstrated that the level of Mon2 subset increased in the first week after STEMI and was associated with worsened outcomes during a 2.5-year follow-up period [83]. These findings are in agreement with the results from another study [28] showing increased levels of Mon2 monocytes in patients with worse CHF. Thus, the Mon2 subset may take part in the healing process after MI since this is the only known subset capable of promoting angiogenesis during the healing process after MI [28]. Moreover, Mon2 was the only subset that increased in patients with stable HF, and the amount further increased under acute HF [84]. Notably, a high Mon2 count was associated with better survival during the study. Therefore, Mon2 monocytes may have potentially protective properties in patients with failing hearts. Mon2 could also be related to acute inflammation. Moreover, it seems that these monocytes are not desirable, and may even be harmful, after the acute phase. Thus, it is important to elucidate what determines an increased Mon2 count after the acute period of disease in some patients and a decrease in others.

Assessment of the monocyte subset distribution according to the different etiologies of HF showed no statistically significant differences when percentages of subsets were considered [73]. Interestingly, a statistically significant difference was observed in the Mon3 subset when the number of cells was determined (cells/μL). Moreover, Mon3 monocytes showed a significant protective association with all-cause death [73]. Moreover, the number of Mon 3 monocytes was found to be unchanged or reduced in the first week after STEMI [83] and increased in CHF [6,73] even after acute exercise [77]. In a specific comparison related to the levels of this monocyte subset, valvular patients (patients with CHF and valve defects) showed lower percentages (6.7 ± 3.4 versus 8.2 ± 4, *p* = 0.04) and numbers of cells (35.2 (23.7–63) versus 49.1 (34.7–70), *p* = 0.01) than ischemic patients and patients with dilated cardiomyopathy (8.3 ± 3.3, *p* = 0.03 and 49.7 (36.5–77.3), *p* = 0.006, respectively) [73]. Notably, there were no differences observed in the level of the Mon2 subset between CHF etiologies. However, a statistically higher percentage of Mon3 monocytes was found in deceased patients [73].

It is worth noting that the amount of Mon1 monocytes in patients with ischemic HF was close to the values observed in patients with coronary artery disease without HF (the control group) [84]. However, the level of Mon1 increased during HF decompensation. Mon1 monocytes are also known to be involved in myocardial remodeling at sites of dying cardiomyocytes.

Notably, the presented studies on the distribution of monocyte subsets in CHF possess limitations related to the following factors: (i) small numbers of patients; (ii) unequal sizes between analyzed groups, and (iii) incomplete description of comorbidities that might be responsible for the abnormal release of monocytes. Moreover, it is complicated to compare the results because of different causes of CHF and different patient conditions between studies. Nevertheless, it is suggested that Mon2 monocytes predominate in the presence of surviving HF and that the cytokines and chemokines these monocytes release lead to the fibrosis of healthy heart tissue. Thus, as a result, the left ventricular relaxation in diastole is impaired. In contrast, Mon1 monocytes are found to be involved in myocardial remodeling at the site of dead cardiomyocytes. However, it is also possible that a different subset of monocytes involved in myocardial remodeling changes over time with different causes of heart failure and differences in pathogenesis. Thus, the monocyte distribution in patients with different CHF causes and conditions is still not yet well understood. There is, moreover, a lack of knowledge about differences in the monocyte subset distribution of HF caused by idiopathic cardiomyopathy, hypertension, obesity, ischemic heart disease and other factors. More complete knowledge about the differences in monocyte subtypes depending on the severity of CHF, and how these subtypes change following NYHA functional class changes, is also required. The role of sex in the formation of monocyte subsets in both healthy and CHF individuals also remains unclear.

### 4.2. Influence of Monocyte-Secreted Cytokines and Inflammatory Readings on HFrEF and HFpEF Development

Monocytes and macrophages were found to be essential to wound healing and tissue repair through angiogenesis, phagocytosis and favorable remodeling of the extracellular matrix [49]. However, prolonged inflammation leads to harmful remodeling [9] when the cells synthesize too much fibroblast and collagen content, promoting the apoptosis of cardiomyocytes [1].

Inflammation plays different roles in the onset and progression of HF in HFrEF and HFpEF [73,85,86,87]. Fibrosis occurs and is differentially managed between these two HF groups. Ischemic heart disease and cardiomyocyte loss lead to HFrEF [4]. It was previously documented that MI and, subsequently, heart muscle necrosis cause systemic and cardiac inflammation, which involves the activation of monocytes [88]. Activated monocytes produce cytokines and chemokines and thus further promote inflammation [88]. Late cardiac remodeling after MI includes the remodeling of both infarcted and non-infarcted myocardia since: (i) the unaffected myocardium strives to compensate for the function of the impaired heart area; (ii) the damaged myocardium is replaced by a collagen scar, and (iii) this scar expands into the healthy area [15]. In addition, the TNF-α secreted by monocytes triggers uncontrolled oxidative stress, cardiomyocyte apoptosis, and even tissue necrosis [89,90]. The loss of cardiomyocytes contributes to the deterioration of heart muscle contractile functions and thus to the development of HFrEF [91]. Moreover, the excessive and prolonged infiltration of monocytes/macrophages into the damaged myocardium causes harmful inflammatory responses that can lead to cardiac fibrosis and adverse myocardium remodeling when LVEF becomes reduced and is insufficient to provide tissues with the necessary supplements and oxygen [92].

Chronic hypertension, cardiomyopathy, and valvular heart disease alter the metabolism in cardiac tissue and can cause HFpEF [93]. It was proposed that HFpEF can be regarded as low-grade chronic systemic inflammation featuring an activated nuclear factor kappa B (NFkB) pathway and the synthesis of pro-inflammatory cytokines and chemokines [93]. The released molecules subsequently activate hematopoietic cells in the bone marrow and spleen, which leads to low systemic inflammation and an increased number of blood leukocytes, neutrophils and monocytes [94]. Furthermore, Mon1 monocytes enter the heart tissue and become the pro-fibrotic macrophage subset (M2) [66], which activates fibroblasts. The activated fibroblasts then synthesize more collagen and fibronectin, which leads to increased myocardial stiffness [4,94]. In this case, the LVEF is normal, but the diastolic function becomes impaired through the prolonged LV relaxation and filling, increased diastolic stiffness, and elevated LV end-diastolic pressure [95]. It is thought that LV stiffness is caused by reduced Ca^2+^ signaling and titin modifications [93]. Notably, cardiac stiffness leads to extracellular matrix changes, cardiac fibrosis, and hypertrophy of cardiomyocytes. These hypertrophic changes result in diastolic dysfunction [96].

The functional diversity of monocytes and macrophages and their ability to contribute to different cardiac processes depend on phenotypic plasticity [97]. However, it remains unclear how the balance of monocyte subsets in both HFrEF and HFpEF is achieved. It seems that certain cytokines and chemokines are factors that determine monocyte subsets and clinical outcomes. For instance, 1.3- to 2.4-fold increased systemic levels of inflammatory markers (TNF-α, IL-6) and chemokine CCL2 were observed in worsening HFpEF compared to stable disease, suggesting that intensified inflammation may contribute to clinical worsening in HFpEF patients [98,99]. Moreover, the twofold increased level of macrophages in myocardial biopsies from HFpEF patients and the 59% stimulated expression of pro-fibrotic cytokine transforming growth factor beta (TGF-β) (compared to control) were associated with fibroblast activation and the excess deposition of collagen [94,100]. Importantly, HFpEF patients also had two- to four-fold elevated circulating levels of neutrophils and Mon1, Mon2, and Mon3 monocytes, while the levels of circulating lymphocytes were not affected [94,97]. These results suggest the development of chronic inflammation during HFpEF.

Another study showed that the seven-day incubation of primary monocytes from healthy subjects in cell media containing 10% serum from HFpEF patients stimulated the differentiation of monocytes into IL-10-expressing M2 macrophages [97]. Thus, long-lasting stimulation in HFpEF patients could push emerging macrophages towards a fibrogenic phenotype, thereby promoting myocardial collagen deposition and diastolic dysfunction. Notably, the synthesis of IL-10 was found to be beneficial in heart tissue repair and the resolution of inflammation following acute injury, thus preventing HFpEF after MI [101,102]. In contrast, IL-10 was found to evoke adverse effects in chronic conditions by promoting myocardial fibrosis and diastolic dysfunction in HFpEF [94]. The fact that the same pathways could result in a positive outcome in HFrEF but could lead to pathology in HFpEF should be kept in mind when designing novel therapeutic strategies to limit disease progression in HF with diverse etiologies.

It was also shown that among HFrEF patients separated into two groups according to neutrophil count (relatively low and high), the CRP and fibrinogen concentrations and monocyte count were higher in the group with a higher neutrophil count [6,81]. These observations are in line with the low inflammatory environment observed in HFrEF patients. Regardless of the cause of HFrEF, inflammation results in cardiac remodeling evoked by cardiomyocyte damage and loss due to cardiomyocyte autophagy, apoptosis and necrosis [6]. In addition, metabolic risk factors in HFpEF occur under chronic low systemic inflammation together with the stimulated expression of adhesion molecules on the endothelial cells, which lead to systemic and local inflammation. Furthermore, the circulating levels of pro-inflammatory markers (IL-6, TNF-α) and acute inflammatory CRP were found to be higher in HFpEF than in HFrEF [98,103]. It was recently found that in HFrEF patients, the monocyte percentage and count were statistically significantly the highest in the NYHA IV group, while the NYHA functional class correlated with the total monocyte count and percentage (r = 0.172) [80]. In addition, the CRP concentration correlated with NT-proBNP (r = 0.203) [80]. Therefore, it can be assumed that the more affected the heart muscle, the stronger the pro-inflammatory environment in patients with chronic HFrEF.

Compounds released into the environment from dying cardiac cells stimulate M2 macrophages (arising from Mon1) to produce anti-inflammatory cytokines, including IL-10 and TGF-β, which preserve neighboring tissue and cardiac functions. Dying cardiomyocytes also secrete damage-associated molecular patterns (DAMPs), including double-stranded DNA. The recognition of DAMPs by M1 macrophages promotes the secretion of pro-inflammatory cytokines, including IL-1β, and leads to collateral tissue damage, adverse ventricular remodeling, and systolic dysfunction [104]. Over months to years, systemic neuroendocrine activation and compensatory mechanisms such as LV wall thinning and chamber dilation lead to HFrEF and its progression [105].

It is also known that in HFrEF patients, the levels of IL-1β and TNF-α can increase two- to six-fold compared to the levels in control subjects, thus signaling worse outcomes [106,107]. Therefore, it is proposed that macrophage-mediated inflammation plays a crucial role in HFrEF pathogenesis. Moreover, failure to clear apoptotic cardiomyocytes can lead to secondary necrosis and the release of DAMPs, which further stimulate pro-inflammatory reactions and collateral tissue injury. Therefore, the increased population of cardiac M1 macrophages in the ischemic heart and persistent inflammation transform the hematopoietic compartment and lead to further macrophage infiltration into the heart, causing harmful remodeling, systolic dysfunction and HFrEF progression (Table 3).

Furthermore, macrophage migration inhibitory factor (MIF; the inflammatory cytokine) mediates the pro-inflammatory effects that lead to fibrotic remodeling in HF due to non-ischemic cardiomyopathy with reduced LVEF. Furthermore, MIF expression is statistically significantly correlated with the degree of myocardial fibrosis (r = 0.51) [108]. The main differences between HFpEF and HFrEF in monocyte and macrophage subsets, pathogenesis, and myocardial changes are presented in Table 3.

Overall, there is still an ongoing debate as to whether the functions of monocytes and macrophages should be regarded as causes or consequences in human CHF development. Despite the findings that Mon2 is more important in HFpEF and Mon1 in HFrEF, further investigations are needed to define the interchange of signals between macrophages and other cardiac resident cells such as monocytes, fibroblasts, and cardiomyocytes. Moreover, the influence of comorbidities (diabetes mellitus, anemia, respiratory diseases, arrhythmias, and others) and risk factors (such as obesity, hypertension, and myocarditis) on the levels of monocyte and macrophage subsets and their functions must also be determined to revise existing therapeutic strategies. Thus, the choice of inflammatory cytokines as a therapeutic target and the monocytes themselves necessitate more research on this topic, as existing studies are not sufficient to prove certain targets for the treatment under consideration.

## 5. Conclusions

According to the available literature data, it can be suggested that monocytes and macrophages take part in both acute and chronic HF processes. Moreover, every patient, depending on the severity of heart tissue damage and the general condition of the body, reacts differently by releasing different cytokines, which subsequently leads to specific organism responses, including the formation of different monocyte subsets, the formation of specific subsets of macrophages, and the release of certain additional cytokines. Finally, these processes lead to the inhibition or maintenance of inflammation in the damaged heart tissue. Consequently, whether a patient’s condition improves or worsens depends on whether the inflammation is suppressed or sustained. Therefore, elucidation of the mechanisms of pathogenesis would help medical professionals direct the patient’s immune system only towards healing. Overall, there is still an open question regarding the pathways of monocyte subset formation. Moreover, Mon1 monocytes are characterized by pronounced phagocytosis and the ability to differentiate into macrophages and synthesize pro-inflammatory cytokines. Thus, Mon1 monocytes are involved in myocardial remodeling at the sites of dying cardiomyocytes. Mon2 monocytes are involved in apoptosis regulation and angiogenesis and secrete both anti- and pro-inflammatory cytokines, whereas the Mon3 monocytes express anti-inflammatory potential and patrol the endothelium. In addition, it is suggested that the Mon2 subtype predominates over the other two subtypes under surviving CHF conditions, whereas Mon1 monocytes dominate in HFpEF patients, and the Mon3 subtype predominates in HFrEF patients. However, the distribution of various subtypes in patients with different CHF conditions is still obscure, and further research is certainly needed.

## Figures and Tables

**Table 1:** The subsets of human blood monocytes with certain surface markers, produced specific cytokines, activated physiological functions, distribution in the blood and implicit locations of subset formation.

	Classical (Mon1) Subset CD14^++^/CD16^-^	Intermediate (Mon2) Subset CD14^++^/CD16^+^	Non-classical (Mon3) Subset CD14^+^/CD16^++^	Reference
Highly expressed surface markers	CCR1, CCR2, CD1d, CD9, CD11b, CD33, CD36, CD62L, CD64, CD99, CLEC4D, CLEC5A, CXCR1-4	CCR5, CD11b, CD32, CD40, CD47, CD54, CD64, CD80, CD86, CD163, GFRα2, HLA-ABC, HLA-DR, TNFR1	CD45, CD97, CD116, CD123, CD294, CD11c, CX3CR1, P2RX1, Siglec10, SIRPα, SLAN, TNFR2	[19,20,21,22,23,24,25,26,27,28,29,30,31,32,33,34,35]
High levels of cytokines	IL13Rα1, G-CSF, CCL2, MCP-1	IL-6, IL-8, IL-10, TNF- α	TNFα, IL-1β, IL-6, IL-8	[21,27,36,37]
Activated function	Phagocytosis; adhesion to the endothelium; migration; anti-microbial responses; inflammation	Antigen presentation; participation in proliferation and inflammatory responses; regulation of apoptosis; trans-endothelial migration; high ROS production	Complement and FcR-mediated phagocytosis; trans-endothelial migration; adhesion; anti-viral responses; patrolling the endothelium	[20,38]
Part of total monocyte count in the blood (%)	80.1 ± 7	3.7 ± 2	6.2 ± 2.8	[20,26,37]
Implicit place of formation/persistency	Bone marrow/tissues	Peripheral blood flow or tissues/blood	Peripheral blood flow or tissues	[39,40]
Lifespan	1 day	3–4 days	4–7 days	[41]

CD14: a glycosylphosphatidylinositol (GPI)-anchored receptor known to serve as a co-receptor for several Toll-like receptors (TLRs) both at the cell surface and in the endosomal compartment; CD16: a type I transmembrane low-affinity receptor for IgG (FcγRIIIa); CD36: a class B scavenger receptor; CCR2: C-C chemokine receptor type 2 (CD 192); HLA-DR: one of the key cell surface molecules expressed on antigen-presenting cells; CD11c: a type I transmembrane protein expressed on monocytes, granulocytes, a subset of B cells, dendritic cells and macrophages; CXCR1: one of more than 20 distinct chemokine receptors, a receptor to interleukin-8; CXCR2: a member of the chemokine receptor family involved in neutrophil chemotaxis; CLEC4D: a member of the C-type lectin/C-type lectin-like domain (CTL/CTLD) superfamily with diverse functions, such as cell adhesion, cell-cell signaling, glycoprotein turnover and roles in inflammation and immune response; CLEC5A: a pattern recognition receptor for members of the Flavivirus family; CD40: a receptor also known as TNFRSF5, a tumor necrosis factor receptor superfamily member 5; IL13RA1: interleukin 13 receptor subunit alpha 1; CD62L: L-selectin; CD86: a type I membrane protein, which is a member of the immunoglobulin superfamily; CLEC10A: Ca^2+^-dependent lectin-type receptor family member 10A; CD301, an endocytic receptor; CD99: a cell surface glycoprotein; GFRA2: a glial cell line-derived neurotrophic factor receptor alpha 2; CD163: an acute phase-regulated and signal-inducing transmembrane receptor for the hemoglobin–haptoglobin (Hb:Hp) complexes; CD74: a cell-surface receptor for the cytokine macrophage migration inhibitory factor; P2xR1: purinoceptor subunit; CD1d: a glycoprotein and a member of the CD1 family of Ag-presenting molecules; CXCR4: a G-protein-coupled chemokine receptor for extracellular ubiquitin; G-CSF: granulocyte colony-stimulating factor; IL: interleukin; LPS: lipopolysaccharide; MCP-1: monocyte chemoattractant protein 1; CCL2 and CCL3: small cytokines that belongs to the CC chemokine family.

**Table 2:** The distribution of monocyte subsets in CHF.

Investigated Person		CHF (IDC (65% of Investigated Population) and ISH)	Healthy	Ambulatory Treated CHF I-IV NYHA Functional Class		CHF I-III NYHA Functional Class, 57% ISH, 43% IDC	Healthy	Stabile CVD where LVEF > 43%	Healthy	Systolic CHF II-IV NYHA Functional Class	Healthy
				Alive	Deceased						
Reference		[77]		[73]		[78]		[27]		[79]	
n		20	15	293	107	30	26	14	13	59	29
Gender F/M		7/13	6/9	80/213	29/78	M	M	5/9	8/5	14/45	14/15
Age		51,2 (9,3)	43,5 (5,0)	66,7 (11,9)	76,9 (9,7)	70,9 (2,1)	69,5 (2,2)	60 (9)	59 11)	58,1 (13,9)	59,7 (6,4)
BMI		26,6 (3,8)	24,2 (2,3)								
Exclusion criteria /Inclusion criteria		Active inflammatory or malignant disease and treatment with immunosuppressive agents /CHF patients		Active inflammatory disease /HF irrespective of etiology (at least 1 HF hospitalization or reduced LVEF)		Inflammatory, cancer, autoimmune diseases, malnutrition /CHF lasting longer than 1 year, clinical stability and the same treatment in the last 3 weeks, LVEF≤45%		ACS or coronary revascularization within the last 6 months, current inflammation within the last 6 months, autoimmune or malignant diseases, dialysis-requiring renal failure /stable CAD (1–3 vessel disease)		Acute heart failure or acute coronary syndrome, or haemodialysis, or known systemic inflammatory disease /LVEF<40%, no recent cardiac decompensation	
Leukocyte count (10^6^/mL)		**8.24 (1.82)**	**7.17 (1.60)**			**8.34 (0.62)**	**6.45 (0.26)**	7.0 (4.2–9.4)	6.7 (4.3–15.6)		
Monocytes	% of leukocytes	**7.72 (1.88)**	**6.28 (1.24)**					**5.1 (3.6–10.8)**	**3.7 (3.2–8.0)**		
	Count (cells/µL)	**628 (159)**	**450 (128)**			**629 (61)**	**509 (34)**	**354 (131–452)**	**308 (187–440)**		
Monocyte subsets (% of monocytes)	% Mon1	87.34 (3.54)	88.09 (4.73)	50.4 (16.5)	48.9 (19.08)					**73.5 (1.8)**	**84.3 (1.9)**
	% Mon2	4.74 (2.46)	4.51 (2.05)	41.2 (16.5)	44.0 (18.8)	**12.3 (8.7–14.8)**	**5.9 (4.7–6.9)**				
	% Mon3	7.92 (2.19)	7.39 (3.17)	**8.42 (4.0)**	**7.1 (4.0)**						
Monocyte subsets (cells/L)	Mon1	**550.3 (143.9)**	**395.2 (107)**	327 (222–435)	363 (227–451)			303 (113–437)	266 (161–412)		
	Mon2	**29.3 (17.1)**	**20.7 (13.5)**	**253 (170–374)**	**303 (186–470)**						
	Mon3	**49.3 (17.3)**	**34.1 (20.9)**	48 (35–71)	44 (27–73)						

The readings with statistically significant differences between the healthy and patient groups are marked in bold. CHF: chronic heart failure, CVD: cardiovascular disease, F: female, M: male, ISH: ischemic heart disease, IDC: idiopathic dilated cardiomyopathy, CAD: coronary artery disease, ACS: acute coronary syndrome.

**Table 3:** The differences between HFpEF and HFrEF in monocyte and macrophage subsets, pathogenesis and myocardial changes [93,98,99,100,101,103,104,105].

	HFpEF	HFrEF
Predominant monocyte subset in the myocardium	CD14^++^, CD16^+^ (Mon2)	CD14^++^, CD16^-^ (Mon1)
Differences in pathogenesis	Low-grade systemic inflammation; monocytes produce chemokines (MCP-1, TNF-α, TGF-β, IL-6).	Cardiac inflammation; fibrosis is associated with monocyte surface TLRs and the migration of Mon1 monocytes into the myocardium due to increased levels of IL-1β and CCR2 expression.
Macrophage subset	M2	M1
Myocardial changes	LV stiffness is caused by reduced Ca^2+^ signaling; conversion of titin into a less flexible form; perivascular and interstitial fibrosis; fibrotic changes in extracellular matrix and cardiomyocyte hypertrophy; impaired relaxation of the heart muscle	Collagen scar formation; cardiomyocyte apoptosis; impaired myocardial contraction

CD14: a glycosylphosphatidylinositol (GPI)-anchored receptor known to serve as a co-receptor for several Toll-like receptors (TLRs), both at the cell surface and in the endosomal compartment; LV: left ventricle; CD16: a type I transmembrane low-affinity receptor for IgG (FcγRIIIa); CD36: a class B scavenger receptor; CCR2: C-C chemokine receptor type 2 (CD 192); TNF-α: tumor necrosis factor α; IL: interleukin; MCP-1: monocyte chemoattractant protein 1 (a key chemokine that regulates monocyte migration); TGF-β: transforming growth factor beta (a multifunctional cytokine).

## Data Availability

Not applicable.
